# New York Letter

**Published:** 1882-04

**Authors:** 


### ARTICLE VII.

NEW YORK CITY, March 16, 1882.

EDITORS OF CHICAGO MEDICAL JOURNAL AND EXAMINER: We are still talking about the new code and the prospective new medical laws. The general opinion concerning the former is that it is a mongrel affair and no great improvement on the old one. It includes homœopaths, but excludes mineral waters. It pretends to be liberal, but inserts the ridiculous prohibition of "interviewing." The fact is that the day of ethical codes is past, and it might just as well have been recognized at the meeting of the State Society. Indeed, Dr. Morse's resolution to abolish the whole came very near passing, and it will doubtless be adopted in a year or two.

Medicine is theoretically a learned profession, but practically it is ruled by much the same laws and instincts as trade. We work for a recompense nowadays—not for an honorarium. It may be unfortunate, but it is true. And why should the doctor need to have his actions regulated by a written code, and not the lawyer or the grocer? We want a short code which will teach young physicians medical etiquette, not medical words.

The journalistic threats that the New York State medical delegation to the American Medical Association will not be received, are preposterous. The delegates represent the foremost medical men in the city. The American Association would receive more injury than it could gain by excluding them. If it did so, it would indeed only make itself ridiculous.

The charge that the movers and formers of the new code wanted homœopathic consultation practice is a silly one. It does not seem to be remembered that our County Medical Society long ago permitted consultation with all regularly-qualified practitioners who did not practice according to a dogma. There are very few dogmatic homeœopaths now. Consultation with the quasi-eclectics has not been an uncommon thing. But furthermore, the homœopaths have got along without us so long that they can still do very well alone.

There is quite an active movement in favor of a law taking the licensing power away from the colleges and giving it to a State Examining Board. The graduation of some six hundred new doctors in the next few weeks emphasizes the interest aroused at the meeting of the State Society concerning this matter. The subject even receives attention from the daily press, which announce that there is now one doctor to every five hundred inhabitants in this country, and that each doctor can therefore average only two or three patients per week. The medical colleges do not openly oppose such a law. Indeed, with the universal sentiment demanding that something be done to protect the profession, they would hardly dare to do so.

The small-pox scare has nearly subsided, although the disease is still present. Very nearly everybody has been vaccinated. The demand for virus from one large drug establishment was one thousand points per week. The amount of free vaccination done at the dispensaries, and elsewhere, has been very great, averaging over a thousand cases a day. I have heard of no fatal results from the bovine virus, though it makes a very sore arm. From what I am told, there is a vast deal of ignorance among the profession on this same subject of vaccination. And there are said to be a great number of non-protective vaccinations. I believe in the anti-vaccination societies most heartily. They are doing a good work—that of forcing the profession to inquire into what vaccination really does. It was not until last January that any records began to be kept at the city small-pox hospital. These records now show that a large majority of the patients received had been vaccinated. And this is what other statistics reveal. The fault, however, is not in the vaccine disease, but in the vaccine virus and the doctor who uses it. In my own experience I have found that one kind of virus supplied by a reputable firm produced no effects, while another kind upon the same persons was active. Many other physicians have told the same story. Yet thousands of quills of this virus have been used here and elsewhere every week.

It is rather the fashion here, in deference to the wish of the patient to get his money's worth, to scrape or scratch deeply so as to make the blood very apparent. This is done considerably in the dispensaries, since the deep method is quicker. My friend, Dr. Derby, who is one of the few experts on vaccination in the city, tells me that it is this deep scarification which is largely the cause of the bad arms, with erysipelas, sloughs, etc., and that the vaccine protection is lessened or spoiled by having these severe inflammations take place. The patient believes the worse the arm the surer the protection, and so does the doctor very often.

At one of our dispensaries here there is a female laryngologist and oculist, Dr. Rosa Welt. She has studied in Germany, and is said to be very accomplished. She is, I believe, the only lady specialist we have in this line, and we hope to be proud of her. The alternate to the same class is Dr. Baruch, who showed me the other day some interesting results of the treatment by insufflation of starch and nitrate of silver powders (B. gr. ii—gr x. to ℥i).

Dr. Baruch is of the opinion that the proper application of powders will, in very many cases, do quite as much for the throat and nose as the most elaborate and powerful spray apparatus. Certainly we should all hope so, for the general practitioner can afford insufflators, and with a little experience can use them as well as a specialist. And he need not send away his cases if all the treatment given by these specialists is based on the simple principles of first cleansing the mucous membrane and then applying astringents.

At the Bellevue outdoor department, Dr. Katzenbach, who is a skillful therapeutist, has told me of a large number of cases in which he gets particularly good results from combining muriate of ammonia with the muriatic tincture of iron. The suggestion is from some English physician whose name I do not recall. It certainly seems to add much to the efficacy of the iron. Indeed, about the only cases in which I have seen unquestionable and direct benefit from this drug have been when the ammonium was given with it. Iron is a good deal like true religion in most cases—it must be taken with a little water and a good deal of faith.

Dr. Wooster Beech, of this city, has invented a very ingenious instrument for removing the spinal cord through the foramen magnum without opening the spinal canal at all. It consists of a long, flat, pliable strip of brass or steel, to which are attached at regular intervals steel rings just large enough to slip over the greatest expansion of the cord. The lower ring has a very sharp, inferior edge. All of the rings are split transversely so that their size can be changed. The brain and medulla are removed, and the instrument slipped down over the cord, cutting the nerves as it goes. When the end is reached, the cord is cut off by a fine wire loop connected with the first ring and running up along the flat slip. If the instrument works as it promises, a great deal of trouble and disfiguration can be saved, and spinal cords will be taken out oftener than they are at present.

The Columbia Veterinary College, and "School of Comparative Medicine," as its authorities like to add, is a rather curious place. I visited it with Dr. Beech, the other day, in order to try his new instrument. We were shown around the establishment. There was a menagerie of dead animals in the dissecting-room. On one table were the remains of a horse; on another the carcass of a camel; near by was the muscular preparation of an antelope, which was being prepared in hope of getting the college anatomical prize. Some horses' legs, the muscular dissection of a dog, and a dead boa constrictor some fifteen feet long were disposed about the place. Several rabbits with the supposed virus of anthrax in them were hopping around in the room above—very robust and not too chaste. The case of the camel was the most interesting one. I examined his stomachs, of which he has five, and the curious "water-bags" in the first and second division, wherein he is supposed to bottle up liquid supplies. The cause of his death was a perinephritis, ending in absolute gangrene of the outer parts of the kidney. Up-stairs, in Dr. Satterthwaite's laboratory, we were shown some beautiful specimen's of the "pearl disease," or bovine tuberculosis." The tubercles, which Virchow considers sarcomatous rather than tuberculous, are in the shape of round, hard, white bodies attached to the pleura and other parts by a pedicle. There were also speciments of contagious pleuro-pneumonia, and of anthrax, the latter appearing in the form of deep, ugly-looking ulcers on the mucous membrane of the stomach. Dr. Satterthwaite, in connection with Drs. Porter and Dana, have lately made some experiments with the inoculation and cultivation of anthrax.

The medical and spiritual charities of the city are just now in a flutter over some very numerous and generous bequests from the late Miss Sarah Burr. This lady died on the 1st inst., worth about $3,000,000, and having very few relatives with whom to leave the money, she accordingly divided up most of it, and there is scarcely a charity in the city which does not receive a portion.

First of all she gives $200,000 to establish a new dispensary, to be called the Good Samaritan Dispensary. Then $30,000 is given to St. Luke's and the same sum to the Woman's Hospital; also the Home for Incurables, the German Hospital, Mt. Sinai Hospital, Home for Consumptives, Society for Relief of the Ruptured and Crippled, get $10,000 each. The Northeastern Dispensary gets over $15,000, and most of the other dispensaries get $5,000. The Training School for Nurses gets $10,000. Altogether, nearly half a million is given to various medical charitable institutions. It is rather curious to find that $10,000 is given to the Jewish Hospital here, and $10,000 more to the Society for the Conversion of the Jews.

Two new medical journals have appeared since I last wrote. One is the *Quarterly Illustrated Journal of Medicine and Surgery.* It is edited by Dr. G. H. Fox, and occupies a peculiar field. The first number contains several colored lithographs of plastic operations performed by Dr. Post. It is in large quarto form, and it can therefore present a certain class of subjects better than is possible in ordinary journals. Dr. McBride's *American Journal of Neurology and Psychiatry* is happily named, and has some especially good features. Its editorial notes and comments are excellent, and it aims to supply the neurological needs of the general practitioner.

The latest literary gossip is, I believe, about the *Journal of Obstetrics.* Its editor, Dr. Mundé, is not so popular among gynæcologists as he might be. Consequently, the Obstetrical Society voted to give its reports to the *New York Medical Journal*, and thus the "foremost obstetrical journal of the country" is left out in the cold. How much injustice is done I cannot say, but it is not likely that the general practitioner will suffer much from the internecine strife. It is said that the house of Lea & Co. is to publish a "Gynæcological Encyclpædia" which will tell everything that there is to be told regarding diseases of women. Everyone is entitled to his hobbies, and one of mine is that gynæ cology is demoralizing the women of our cities. No doubt much physical good is done, but women are brought to that stage when they think no more of having a physician look at the cervix than at their tonsils, while in many cases a morbid craving for local treatment, tampons and pessaries, is generated. That women believe in the efficacy of the speculum may be inferred from an anecdote told of one of our eminent physicians. He examined a lady patient with one of these instruments one day. The exploration ended, he was about to withdraw the speculum. "Excuse me, doctor," said the patient, "but for a long time I have been feeling pains in my stomach. While you are about it, won't you see what is the matter there?"

At the Academy of Medicine this month, Dr. Castle read a practical paper on the treatment of some of the ordinary diseases of women. He recommended very highly the use of Fowler's solution for some of the difficulties, usually thought to be digestive, that occur among women at or near the time of the menopause. He referred to the effect of the overdistension of the abdomen with flatus as a cause of prolapse. This idea he claimed as original. He also made a useful suggestion in regard to giving the vaginal douches. The simplest way was to have the patient in a sitz-bath. At a previous meeting of the Academy in February Mr. Wright read a paper on plumbing and allied topics. It was not, I am told by experts, a very accurate or novel contribution, but it seemed to interest the audience, who probably were as ignorant as most doctors are everwhere about plumbing. The discussion brought out, at a subsequent meeting, a paper from Dr. Frank H. Hamilton on "The Struggle for Life Against Civilization and Æstheticism." The tenor of thought is indicated by the title. The paper was rather a novel one for a medical society. It was a thoughtful one, however, and was well discussed.

The subject of Guiteau's insanity was debated at a meeting of the Medico-Legal Society this month. It was introduced by a paper from Dr. Hammond, which is already in print. The discussion was all one way as regards the question of sanity or insanity, but was rather inharmonious otherwise. Some thought that Guiteau was born insane; some that he became insane when at Ann Arbor. Most seemed to think him a moral idiot, but others thought him a religious monomaniac and with a very fair amount of moral sensibility. Hammond thought he should be hung. Beard intimates that if we hang him we make fifty millions of Guiteaus. The general opinion seemed to be against Hammond's view that some insane are responsible, or at least punishable. It would not be easy to get a clear idea of what our New York experts think of Guiteau's mental condition from the discussion at this society, which, by the way, includes but few, if any, of those connected with the asylums of the city. The view taken by most was that Guiteau was congenitally devoid of moral sense. With this moral vacuity were some intellectual peculiarities characteristic of monomania. It would not be claimed, I

presume, that persons with no moral sense are necessarily insane, since our prisons are full of such, and they are not so rare in the ordinary walks of life. Besides, it would be hard to show that Guiteau was without moral sensibilities. It must be the existence of some intellectual vagaries that would make him out insane. The "parallel" historical cases cited in the discussion were parallel or not according to the listener's standpoint, and were of no logical or argumentative value. The best collection of evidence as to Guiteau's insanity is, it seems to me, in Dr. Beard's article on the subject in the January number of the *Journal of Nervous and Mental Disease.*

At a subsequent meeting of the Medico-Legal Society the paper by Dr. Johnson, of Brooklyn, on anæsthetics, was discussed. There was little said which did not confirm the several conclusions reached by Dr. Johnson, and which are so important that I append them:

1. Anæsthetics do stimulate the sexual functions; the ano-genital region being the last to give up its sensitiveness. Charges made by females under the influence of an anæsthetic should be received as the testimony of an insane person is. It cannot be rejected, but the *corpus delicti aliunde* rule should be insisted on. Dentists or surgeons who do not protect themselves by having a third person present do not merit much sympathy.

2. Deaths from administration of chloroform after a felonious assault, unless the wounding were an unmistakably fatal one, reduces the crime of the prisoner from murder to a felonious assault.

3. The surgeon has no right to use chloroform to detect crime, against the will of the prisoner. But the army surgeon has the right to use chloroform to detect malingerers.

4. The medical expert, notwithstanding he is sent by order of court, has no right to administer an anæsthetic against the wish of the plaintiff in a personal damage suit, to detect fraud.

5. Gross violations of the well-known rules of administering anæsthetics, life being lost thereby, will subject the violator to a trial on the charge of manslaughter.

6. A surgeon allowing an untrained medical student to administer anæsthetics, life being thereby lost, will subject the surgeon himself to a suit for damages. What he does through his agent he does himself.

7. The physician who administers an anæsthetic should attend to that part of the business and nothing else. He should have examined the heart and lungs beforehand. He should have the patient in the reclining position, with his clothes loose, so as not to interfere with respiration; should have his rat-tooth forceps, nitrite of amyl and ammonia, and know their uses, and when to use them and how to perform artificial respiration.

8. In operations on the ano-genital region and the evulsion of the toe-nail, complete loss of sensation in these parts should never be allowed, and no operation on these parts at all should be had under an anæsthetic, unless by the approval of a full consultation who have a knowledge of the dangers.

9. Chloroform cannot be administered by a person who is not an expert to a person who is asleep without awaking him. Experts themselves, with the utmost care, fail more often than they succeed in chloroforming adults in their sleep.

Dr. W. J. Morton gave some statistics showing the comparative danger of chloroform. He thought that its use should be forbidden by law. Dr. Gordon related some experiments in which attempts to chloroform sleeping persons were made unsuccessfully.

NEW YORK CITY, March 16, 1882.

